# Network Analysis of Time Use and Depressive Symptoms Among Emerging Adults: Findings From the Guizhou Population Health Cohort Study

**DOI:** 10.3389/fpsyt.2022.809745

**Published:** 2022-04-01

**Authors:** Zhihao Ma, Fouxi Zhao, Yiying Wang, Tao Liu, Naipeng Chao

**Affiliations:** ^1^Computational Communication Collaboratory, School of Journalism and Communication, Nanjing University, Nanjing, China; ^2^Prevention and Control Institute for Chronic Non-communicable Diseases, Guizhou Provincial Center for Disease Control and Prevention, Guiyang, China; ^3^School of Media and Communications, Shenzhen University, Shenzhen, China

**Keywords:** time use, screen time, time displacement, depression, emerging adult, network analysis

## Abstract

**Background:**

To date, the relationship between diverse time use behaviors and depression status among emerging adults have not been disentangled in the literature. Therefore, if and how the time displacement mechanism activates depressive symptoms among emerging adults remains unclear.

**Methods:**

To fill this gap in the literature, we employed a network analysis to make estimations. The emerging adult sample (*N* = 1,811) was collected by the Guizhou Population Health Cohort Study. Time use behaviors were measured by an adaption of the self-administered International Physical Activity Questionnaire, and depressive symptoms were assessed using the 9-item Patient Health Questionnaire (PHQ-9).

**Results:**

The results revealed that the time displacement mechanism of emerging adults differed from that of adolescents. Sleep duration was not crowded out by other activities, while the time spent on computer use was found to be negatively related to time spent on heavy work activities. Moreover, computer use behavior triggered three depressive symptoms (“Anhedonia,” “Guilt,” and “Motor”), but inhibited “Suicide.” The results of the directed acyclic graph revealed that females and heavy drinkers were at risk of depression.

**Limitations:**

The study sample was confined to only one province, which may limit its generalizability. The cross-sectional design impeded the ability to draw causal inferences.

**Conclusion:**

Our results enhance the current understanding of the internal mechanism of how time use behaviors influence depressive symptoms among emerging adults.

## Introduction

Existing literature have established the effects of physical activity (1, 2), screen time (3, 4), and sleep duration (5, 6) on the depression status of both, adolescent and adult populations. Moreover, previous studies revealed that unhealthy time use activities (e.g., excessive sedentary time, inadequate sleep duration) were risk predictors of physiological diseases, including cardiovascular diseases (7–9), hypertension (10, 11), and cancers (12–14). Based on plentiful existing evidence, the World Health Organization (WHO) and other health institutes proposed updated time use guidelines to direct people’s daily activities for substantial health benefits (15–18). For example, WHO recommended that adults (aged 18–64 years) should do at least 150–300 min of moderate-intensity aerobic physical activities; or at least 75–150 min of vigorous-intensity aerobic physical activities; or an equivalent combination of moderate- and vigorous-intensity activities throughout the week (15). The Canadian Society for Exercise Physiology recommended that adults (aged 18–64 years) should do at least 150 min of moderate to vigorous aerobic physical activities per week, and get 7–9 h of good-quality sleep (17).

However, the existing research pool still has two significant gaps. First, the number of studies focusing on emerging adults, who are undergoing a unique stage of psychological development, and present different mental health features than adolescents and adults older than thirty, are relatively few. Second, previous studies that adopted a regression analysis approach were unable to disentangle the underlying linkages among diverse time use behaviors and depressive symptoms.

Emerging adults are those individuals who are leaving adolescence behind to experience young adulthood, a definition originally proposed by Arnett (19). The concept of emerging adulthood describes a period of development during which an individual has already passed through adolescence, but has not entirely taken on adult responsibility and independent decision making (19, 20). The emerging adulthood stage was initially defined as the age group 18–25 years (19); this was later revised to the age group 18–29 (20). During emerging adulthood, individuals are already biologically mature, but most have not yet established a stable structure in diverse domains of life (e.g., intimate relationships, work, and fertility). These individuals are not identified as socially mature, and thus, they present different behaviors and psychological patterns than either adolescents or adults in their thirties. Previous studies have expounded on the unique patterns observed in emerging adults in several areas, such as substance use (21), Internet addition (22), technology adoption (23), and social integration (24). Previous research revealed that peak alcohol consumption and drug abuse occur during emerging adulthood (25, 26), and some instances of excessive substance use appear to be normative behavior for emerging adults (25, 27). Moreover, one recent study reported that emerging adults present the highest online social network usage among all age groups (28). They have a significantly higher likelihood of adopting pathological social network use behaviors, which further decreases self-regulation, escalates the depression status, and magnifies the likelihood of involvement in cyberbullying (29, 30). However, it remains unclear as to how diverse time use behaviors affect depressive symptoms among emerging adults.

Studies focusing on adolescents provide potential theoretical mechanisms to understand the relationship between time use and depressive symptoms in emerging adults. Previous research has identified that screen time is a crucial predictor of depression (4, 31, 32). Based on this point, Boers et al. (33) put forth three explanations: time displacement, social comparison, and reinforcing spiral. Time displacement refers to the time required for healthy activities (e.g., physical activity and sleep) that may be displaced by excessive sedentary activities, such as screen time (34), causing potential depressive reactions (31, 35). Social comparison states that one’s self-esteem may be damaged by focusing on favorable objects (e.g., having an “ideal body shape” or a “luxury lifestyle”), which triggers relative deprivation, a significant predictor of individual psychological wellbeing (36, 37). A reinforcing spiral implies a selective exposure scenario that is reinforced by both individual intention and algorithmic recommendation of specific media content. Individuals are thereby repeatedly exposed to certain types of content, and depressive reactions might be triggered if individuals view excessive volumes of content that may lead to depression (33). Among these three theories, the social comparison and reinforcing spiral mechanisms were mostly used to understand the negative effects of media on children and adolescents (38–41). Given that emerging adults present a more mature decision-making pattern during daily activities (19), they are relatively less sensitive toward media contents compared with children and adolescents. Thus, we believe that time displacement mechanism is an optimal approach for interpreting the relationship between time use and depression in emerging adults.

Moreover, life course theorists have indicated that behaviors or experiences during one’s early adult life may have potential effects on one’s later life, and such effects tend to accumulate (42, 43). For example, adverse experiences during childhood were found to be crucial risk factors in experiencing psychotic symptoms and health burden (44, 45). However, one recent study demonstrated that individuals who experienced traumatic events during emerging adulthood reported worse health status compared with individuals who only experienced traumatic events during adolescence (46). Such cumulatively disadvantageous phenomena were reported in diverse age groups (47–49). But the cause of the disadvantages was rarely discussed. Therefore, understanding the time displacement mechanism in terms of diverse time use behaviors, and its impact on depressive symptoms during emerging adulthood also has enormous potential for predicting long-term health outcomes.

Existing studies also have several limitations as they have been unable to clarify the detailed patterns of relationship between diverse time use behaviors and depressive symptoms. First, both psychiatric and psychological studies usually presuppose one specific disorder as a latent structure model, with several symptoms as observed variables (50). The latent structure model implies that related symptoms are mutually independent; however, this approach ignores the inter-trigger mechanism among these symptoms (51, 52). Moreover, studies that adopt the traditional regression approach often use the sum score or mean score of a group of symptoms to represent a certain disorder (50). Although this operation is effective in screening for the prevalence of a certain disorder, it neglects the occurrence of specific symptoms in non-disordered individuals. Additionally, most time use studies that adopt a regression approach can only consider one dependent variable in one model, which also presents limitations in simultaneously revealing the interrelationships among diverse time use behaviors.

The network analysis approach was employed in this study to address the methodological gap described above. This approach presupposes that a mental disorder is a complex system, in which the relationships and network properties of different symptoms are identified in detail (51, 53). Both the inter-trigger mechanisms among different symptoms, and the relationships between external shocks and psychiatric reactions can be modeled when using the network analysis approach (54–56). Recent studies have also adopted network analyses to identify the central domains of a specific psychological construct (57), and the central symptoms within a certain mental disorder (58, 59). Moreover, the network analysis approach provides novel insights in understanding the predictability of a certain symptom within a complex network (60, 61), and further identifying the potential interventions of clinical practices (60). Additionally, the network analysis approach was also employed to investigate the issue of comorbidity among different disorders (62). It has the methodological advantage of distinguishing between the bridge symptom and shared symptom among diverse mental disorders (63).

In the current study, both time use behavior and depressive symptoms were complex systems. We employed the network approach in three ways: First, it was difficult to highlight the intercorrelation pattern of diverse time use behavior—namely, the time displacement, using the traditional linear regression approach. Whereas, network analysis has the merit to illustrate the intercorrelation pattern. Second, diverse time use behaviors were conceptually external shocks that may trigger depressive symptoms via different paths which should be calculated via the network analysis. Third, one type of network method—the Bayesian network, provides a novel approach to algorithmically characterize cross-sectional data as a causal system (64). We thus used the Bayesian network to present the potential causal predictors of depression among emerging adults.

Considering the two literature gaps described, the current study employed network analysis to answer the following two questions: (1) Does the time displacement mechanism explain the inter-correlations among diverse time use behaviors in emerging adults? (2) Does the time displacement mechanism explain the connections between time use behaviors and depressive symptoms in emerging adults?

## Materials and Methods

### Participants

The data used in this study were obtained from the Guizhou Population Health Cohort Study, a prospective community-based cohort in Guizhou Province, China. Based on a multistage proportional stratified cluster sampling method, a total of 9,280 adult residents of 48 townships in 12 districts in Guizhou Province were included. The original study took place from 2010 to 2012. The inclusion criteria were: (1) age 18 years or above; (2) living in the study region, and having no plan to move; (3) completing the survey questionnaire and blood sampling; and (4) providing written informed consent. For the current study, we exclude 7,408 participants who were 30 years old or older. Further 38 participants with missing depressive symptom variables, 21 participants with outlier responses on time use variables, and two participants with missing height or weight information were excluded. Finally, the remaining 1,811 participants were eligible for our analysis.

This study was carried out in accordance with the stipulations of the Declaration of Helsinki and approved by the Institutional Review Board of Guizhou Provincial Center for Disease Control and Prevention (No. S2017-02). All participants provided written informed consent at enrollment. The information was collected by trained investigators using a structured questionnaire via face-to-face interviews.

### Assessment Measures

#### Depressive Symptoms

We used the 9-item Patient Health Questionnaire (PHQ-9) to measure participants’ depressive symptoms (65). Participants were asked to rate how frequently they experienced nine specific depressive symptoms during the previous 2 weeks on a 4-point Likert scale, ranging from 0 = not at all to 3 = nearly every day. A higher score for a certain item indicates that participants were experiencing severe symptoms, while a higher total score indicates that participants overall had a severe depressive status. The PHQ-9 used in the current study presented excellent reliability (Cronbach’s alpha = 0.828). The one-factor construct was also supported by the confirmatory factor analyses (CFI = 0.980, TLI = 0.964, RMSEA = 0.057, SRMR = 0.030).

#### Time Use

We measured nine time use behaviors in participants across three domains: physical activities (five items), screen activities (three items), and sleep duration (one item). Time use behaviors were measured by adapting the long version of the self-administered International Physical Activity Questionnaire (IPAQ-L) (66). Measures of time use on physical activities included weekly minutes spent on heavy work activities (vigorous-intensity physical activities during work, farming, and housework), moderate work activities (moderate-intensity physical activities during work, farming, and housework), traffic time (walking or cycling for transport), heavy leisure activities (vigorous-intensity leisure activities such as long-distance running, swimming, and playing football), and moderate leisure activities (moderate-intensity leisure activities such as quick walking and performing Tai Chi). Measures of time use for screen activities included weekly minutes spent on watching TV, using computer, and playing video games. Sleep duration was measured in weekly minutes. We employed confirmatory factor analyses to assess the psychometric properties of the measurement. Results revealed that the one-factor construct had adequate fit indices (CFI = 0.955, TLI = 0.919, RMSEA = 0.035, SRMR = 0.027).

#### Control Variables

We took sex, age, body mass index (BMI), drinking behavior, and smoking behavior as control variables. Age and BMI were treated as continuous variables. Sex, drinking behavior, and smoking behavior were treated as binary variables. Sex was coded as female or not, drinking behavior was coded as heavy drinker (drinking frequency of 3–4 days a week or more) or not, and smoking behavior was coded as daily smoker (who reported smoking every day) or not.

### Statistical Analysis

#### Descriptive Analysis

We first used a descriptive analysis to present the outline of the participants’ data. We employed a cutoff point of five to calculate the prevalence of mild depression among the current study sample (65). Moreover, we conducted a correlation matrix among nine items of time use behavior and the PHQ-9 score to present the basic patterns of potential time displacements, and possible correlations between diverse time use behaviors and depressive status.

#### Network Estimation

When using a network analysis approach, all variables are treated as nodes, and edges among the nodes can be interpreted as partial correlation coefficients among these variables (67). Given that we included both continuous and binary variables in the analysis, we employed a mixed graphic model approach via *R* package *mgm* software (an algorithm of regularized generalized regression) to estimate the networks (68), and we used the extended Bayesian information criterion with tuning parameter γ = 0.5 to make the estimates.

We first estimated a network that only includes time use and symptom items to present the inter-correlations among the diverse items. The second network included time use items, symptom items, and all control variables; which were estimated to verify if the findings in the first network were stable. Networks were visualized using the *R* package *qgraph* software (69). Additionally, to assess the accuracy of the edges in the two networks, we constructed a 95% bootstrapped confidence interval around the edges (67). The accuracy estimation was conducted using the *R* package *bootnet*, and 1,000 resamples were used for the bootstrapping technique. Moreover, the correlation stability coefficient (CS-coefficient) was used to assess the edge stabilities of two estimated networks.

#### Directed Acyclic Graph

To identify potential causal directions among the diverse time use behaviors, depression status, and controlling variables, we adopted the Incremental Association Markov Blanket (IAMB) algorithm, a constraint-based structure Bayesian network learning algorithm implemented in the *R* package *bnlearn* (70), to estimate the directed acyclic graph (DAG).

Following suggestions from an existing study (71), the total score of the PHQ-9 was included in the DAG estimation. We set no whitelist to elaborate on the efficacy of the IAMB algorithm to calculate the edges within the network. Meanwhile, as sex and age were not influenced by other variables, and we also assumed that depression status cannot influence time use behaviors, the following edges were blacklisted: (1) all edges toward sex and age; (2) edges from the total score of PHQ-9 toward nine time use behaviors. Moreover, we performed 1,000 non-parametric bootstraps to check the stability of the DAG results. Based on the bootstrapping results, edges (both directed and undirected) related to the total score of PHQ-9 and crucial time use behavior were re-calculated via *t*-test or correlation test to reveal the causal triggers of depression. All *R* packages were carried out using version 4.1.2 of *R* software.

## Results

### Descriptive Results

Table 1 provides a description of all the variables used in the current study. Of the sample, 49.70% (*n* = 900) participants were female, 22.25% (*n* = 403) were daily smokers, and 5.52% (*n* = 100) were heavy drinkers. The mean age of participants was 24 years (mean = 23.88, SD = 2.309). Participants reported an average healthy BMI (mean = 21.905; SD = ±3.038). The total PHQ-9 score for the sample was fairly low (mean = 0.701; SD = ±1.856), and the percentage of participants with mild depression was 5.02% (*n* = 91). These results indicate that the participants in the current study did not experience significant depressive symptoms. The participants’ mean weekly sleep duration was 3,415.71 min, which meets the recommended sleep time for adults as suggested in previous studies (72). However, the time spent on both heavy leisure activities (mean = 18.771, SD = ±92.525) and moderate leisure activities (mean = 21.526, SD = ± 111.559) was significantly lower than the WHO’s recommendations, which states that adults should get 150–300 min of physical activity per week (15). The mean weekly duration of participants’ heavy and moderate work activities were 270 and 444 min, respectively. On average, they spent 227 min in traffic per week. Their mean weekly duration of watching TV was 883 min, using computer was 347 min, and playing video games was 43 min.

**TABLE 1:** Descriptive statistics (*N* = 1,811).

Variables	Mean (Std. dev.)	*N* (%)	Min	Max
**Depression status**				
Sum score of PHQ-9	0.701 (1.856)	–	0	23
Mild depression (1 = yes)	–	91 (5.02%)	0	1
Time use behaviors (minutes per week)				
Heavy work activities	270.413 (574.878)	–	0	3360
Moderate work activities	444.445 (630.311)	–	0	3570
Traffic time	226.596 (326.428)	–	0	2400
Heavy leisure activities	18.771 (92.525)	–	0	1200
Moderate leisure activities	21.526 (111.559)	–	0	1800
TV watching	882.808 (576.518)	–	0	5040
Computer use	347.368 (666.677)	–	0	5040
Video game	42.591 (195.817)	–	0	3360
Sleep duration	3415.71 (452.301)	–	0	5040
**Control variables**				
Female (1 = yes)	–	900 (49.70%)	0	1
Age	23.88 (3.309)	–	18	29.98
BMI	21.905 (3.038)	–	14.479	37.188
Smoking (1 = daily smoker)	–	403 (22.25%)	0	1
Drinker (1 = heavy drinker)	–	100 (5.52%)	0	1

Table 2 presents the correlation matrix of relationship between time use and the total score of the PHQ-9. To present comparable results, all time use behaviors were standardized. The results revealed that participants who spent more time on moderate work activities reported a lower PHQ-9 score, while participants who spent more time using computer reported a higher PHQ-9 score. The positive relationship between computer usage and PHQ-9 score implies that computer use may function as a direct trigger of depression, and therefore, we should focus on the time displacement mechanism around computer use. Computer use time was negatively correlated with both heavy work activities and moderate work activities; however, it was positively correlated with heavy leisure activities and moderate leisure activities. Additionally, the results revealed that sleep duration was negatively correlated with traffic time and duration of playing video games.

**TABLE 2:** Correlation matrix of relationships among time use and the total score of PHQ-9.

Variables	(1)	(2)	(3)	(4)	(5)	(6)	(7)	(8)	(9)	(10)
(1) Heavy work activities	1.000									
(2) Moderate work activities	0.099 (0.000)	1.000								
(3) Traffic time	0.145 (0.000)	0.203 (0.000)	1.000							
(4) Heavy leisure activities	−0.005 (0.824)	−0.039 (0.098)	0.162 (0.000)	1.000						
(5) Moderate leisure activities	−0.041 (0.084)	−0.021 (0.378)	0.076 (0.001)	0.462 (0.000)	1.000					
(6) TV watching	−0.026 (0.276)	0.045 (0.054)	0.016 (0.500)	−0.042 (0.075)	−0.009 (0.700)	1.000				
(7) Computer use	−0.170 (0.000)	−0.161 (0.000)	−0.036 (0.127)	0.176 (0.000)	0.091 (0.000)	−0.044 (0.061)	1.000			
(8) Video game	−0.036 (0.128)	−0.063 (0.008)	0.025 (0.288)	0.165 (0.000)	0.088 (0.000)	0.072 (0.002)	0.319 (0.000)	1.000		
(9) Sleep duration	−0.038 (0.101)	0.001 (0.952)	−0.049 (0.037)	−0.037 (0.118)	0.030 (0.203)	0.017 (0.460)	−0.028 (0.230)	−0.059 (0.011)	1.000	
(10) Sum score of PHQ-9	0.025 (0.283)	−0.065 (0.005)	−0.013 (0.595)	−0.017 (0.469)	−0.007 (0.766)	0.023 (0.321)	0.086 (0.000)	0.010 (0.657)	−0.017 (0.460)	1.000

*All time use behaviors were standardized; P-values were presented in parentheses.*

### Results of the Network Estimation

**FIGURE 1:**
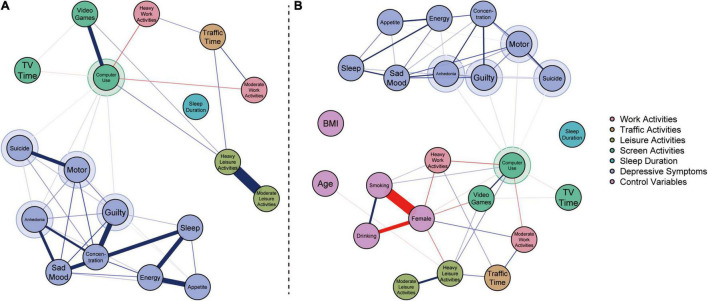
Results of kestimated network models. The blue edges denote the positive relationships, and the red edges denote the negative relationships. The direct linked nodes among time use behaviors and depressive symptoms were highlighted with larger circles. **(A)** Results of the estimated network without control variables. **(B)** Results of the estimated network with control variables.

The estimated network results are shown in Figure 1. Figure 1A displays the internal linkages among diverse time use behaviors and the nine depressive symptoms. Figure 1B displays the results of the network with the control variables. The reference names of items used for the assessment of time use and depressive symptoms are listed in Supplementary Table 1. Detailed edge weights are listed in Supplementary Tables 2, 3, and the bootstrapped accuracy plots are displayed in Supplementary Figures 1, 2. Moreover, two networks have high edge stabilities (all CS-coefficients = 0.75).

After controlling for all time use behaviors and depressive symptoms, Figure 1A shows that the negative relationships between sleep duration and traffic time, and between sleep duration and video games time were not significant. Computer use time is positively correlated with time spent on video games and heavy leisure activities, but negatively correlated with TV watching time, heavy work activities, and moderate work activities. The direct linkages between leisure activities (both heavy and moderate) and work activities (both heavy and moderate) were not significant; however, Figure 1A reveals that negative relationships between leisure activities and work activities were mediated by computer use. Figure 1B presents consistent results after controlling for age, sex, BMI, smoking behavior, and drinking behavior.

According to Figure 1A, computer use was the only trigger for depressive symptoms. Computer use significantly triggered three depressive symptoms: “Anhedonia,” “Guilt,” and “Motor.” These results imply that participants who spend more time using computers will have little interest or pleasure in doing other things in their daily life, may feel bad about themselves, and may have slow behavioral reactions. However, computer use was found to be an inhibitor of “Suicide.” The linkages between computer use and these four depressive symptoms were found to be robust when controlling for age, sex, BMI, and smoking and drinking behaviors (see Figure 1B).

### Results of the Directed Acyclic Graph

**FIGURE 2:**
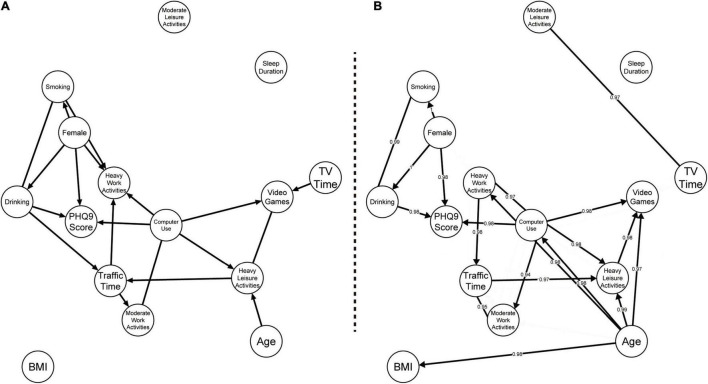
Results of directed acyclic graph (DAG). **(A)** The initial estimated results of the DAG. **(B)** The results based on 1,000 bootstrap replications. Numbers on each edge indicate the non-zero proportions.

Figure 2 presents the results of the DAG. Figure 2A presents the initial estimated DAG results, where computer use has direct effects on heavy work activities, heavy leisure activities, video games time, and the total score of PHQ-9. The total score of PHQ-9 was directly affected by computer use, sex, and drinking behavior.

Figure 2B presents the bootstrapped inclusion proportions of each directed and undirected linkage among the variables. Compared with the results in Figure 2A, predictors of the total score of the PHQ-9 were consistent: female sex (diff = 0.267, *t* = 3.067, *p* < 0.01), being a heavy drinker (diff = 0.242, *t* = 1.268, *p* = 0.205), and computer use (*r* = 0.086, *p* < 0.001) were predictors for the risk of depression.

The direct linkages related to computer use that are presented in Figure 2B were slightly different from the results in Figure 2A: first, the linkage between computer use and heavy work activities was undirected in Figure 2B; second, the linkage between computer use and moderate work activities was directed from computer use to moderate work activities in Figure 2B; third, age was found to directly affect computer use (Figure 2B). Using a *t*-test and correlation test, the DAG results in Figure 2B revealed that older participants reported decreased time spent on computer use (*r* = −0.085, *p* < 0.001). The time spent on computer use was negatively correlated with the time spent on heavy work activities (*r* = −0.170, *p* < 0.001). Moreover, computer use was found to directly decrease the time spent on moderate leisure activities (*r* = −0.161, *p* < 0.001) and increase the time spent on both heavy leisure activities (*r* = 0.176, *p* < 0.001), and on video games (*r* = 0.319, *p* < 0.001).

## Discussion

To the best of our knowledge, this is the first study to adopt a network analysis to disentangle the underlying linkages among diverse time use behaviors and depressive symptoms in emerging adults. While the prevalence of mild depression among emerging adults in the Guizhou Population Health Cohort Study was quite low, this study contributes novel insights to understanding the potential mechanism of triggering depressive symptoms.

First, the time displacement mechanism explains the underlying connections among diverse time use behaviors among emerging adults. Previous studies focusing on adolescents’ time use behaviors revealed a pattern in which higher screen time led to a decreased sleep duration (32, 73). However, our results demonstrated that emerging adults maintain adequate sleep duration, and that it is not influenced by other time use behaviors when controlling for all concerned variables. Poor sleep quality usually leads to significant psychiatric reactions, including inattention and fatigue (74–77). The different patterns of the time displacement mechanism regarding sleep between adolescents and emerging adults could be explained in two ways: First, the crucial stressors that lead to unhealthy sleep among adolescents and emerging adults were different. In China, stressors related to academic work served as crucial risk factors in determining adolescents’ sleep quality (78, 79), while the work environment stressors were key predictors of emerging adults’ sleep quality (80–82). For adolescents, screen time significantly replaces sleep duration (83). However, emerging adult participants in the current study were living in Guizhou—a developing province during the survey time, and they may not have faced significant stressors from the work environment. Second, emerging adults present more mature social interactions and activities compared with adolescents. The time spent on certain behaviors was not compulsory. Thus, as the results revealed that, time spent on computer use was found to be negatively associated with the time spent on work activities. These results indicate that individuals who were undergoing emerging adulthood—a unique developmental stage with initial social independence–replaced their working time with time spent on computer use. The decreased work time is the behavior compensation for excessive screen time.

Second, the time displacement mechanism also explains the connections between time use behaviors and depressive symptoms. The time spent on using computer was significantly correlated with four depressive symptoms. It triggered “Anhedonia,” “Guilt,” and “Motor;” but inhibited “Suicide.” As the connections between time spent on using computers, playing video games, and leisure activities were positive, the negative connection between time spent on computer use and “Suicide” could be explained by the fact that emerging adults usually use computers for recreational purposes, which distracts them from depressive and suicidal content. Given that participants who spent more time on computer use usually spent less time on working activities, the positive connections between computer use and the symptoms “Anhedonia,” “Guilt,” and “Motor” could be because computer use is a potential disengagement coping strategy for emerging adults, to distract them when they have to take on social responsibilities in scenarios they never experienced during their adolescence (84). The disengagement coping strategy granted emerging adults an escape from dealing with the stressors they faced, thus leading to worse depressive status. “Anhedonia” was triggered since emerging adults who used computers excessively may have an inadequate locus of control toward the rewards from the work-related activities, spend more time in online activity (85), and have a high possibility of problematic Internet use (86, 87). Moreover, the activated symptom—“Guilt” could be explained as excessive computer use worsens emerging adults’ social connections (88), and decreased social provisions are typically related to low self-esteem and severe depression status (89). Additionally, the activated “Motor” implies that excessive time spent on computers has potentially negative effects on emerging adults’ physical and cognitive development (90, 91).

Third, the results from the DAG revealed that depression was also linked to two other factors. Sex (specifically, being female) and drinking behavior were risk predictors of depression. While the *t*-test result was insignificant (diff = 0.242, *t* = 1.268, *p* = 0.205), the DAG indicated that being a heavy drinker increases emerging adults’ depression, which is consistent with most previous studies (92–94). This result could be explained by shared genetic and environmental determinant theories (95, 96). Previous twin and adoption studies revealed the presence of genetic influence on alcohol dependence, depression, and the comorbidity of alcohol dependence and depression (95, 97–99). Moreover, such effects were also moderated by social environments, including peer effect (100, 101), living regions (102), and marital status (103, 104). Given that most of the emerging adults have not yet assumed full family responsibilities, the genetic association between heavy drinking behavior and depression status among emerging adults may be intensified by their drinking peers and unmarried status. Additionally, females reported more severe depressive status (diff = 0.267, *t* = 3.067, *p* < 0.01). One previous study indicated that stressors related to pregnancy and postpartum experiences increased the incidence of depression in the female population (105). While not all emerging adult females had experienced pregnancy, fertility-related issues in traditional Chinese family situations may affect females persistently, and lead to further depressive episodes.

Fourth, our results also provide beneficial practical implications to cope with the COVID-19 pandemic. Given the lockdown measure and social distancing recommendations were most adopted policies during the repeated outbreak periods, most of the offline activities have to be taken online. Several depression risk factors, including problematic Internet use (86, 106) and cyberbullying involvement (30, 107, 108) were intensified. Governments and public institutes should promote timely psychological support campaigns to guide individuals’ online behavior, and relieve the stress generated via intensified online activities.

This study has several limitations. First, while the DAG approach provides potential causal directions among the variables, the causal mechanism is obtained by the algorithm, rather than the longitudinal design. The data that were analyzed were cross-sectional, which leads to limitations in causal inference. Second, it remains unknown if the time displacement mechanism has long-term effects on individuals’ depression status. Further studies should collect longitudinal data to address these issues. Third, the sample was recruited from only one province in China. Therefore, caution should be exercised when generalizing the findings to other populations. We hope that scholars, in future, will employ network analysis to test the linkages between time use behaviors and depressive symptoms for other populations. Further meta-analyses of these potential network studies are required. Finally, we only verified the effect of the time displacement mechanism in depression. If and how the other two mechanisms, namely social comparison and reinforcing spiral (33), could explain the depression pathogenesis among emerging adults remains unclear. Further studies are needed to verify how these potential mechanisms influence emerging adults’ mental outcomes.

## Data Availability Statement

The raw data supporting the conclusions of this article will be made available by the authors, without undue reservation.

## Ethics Statement

The studies involving human participants were reviewed and approved by the Institutional Review Board of Guizhou Provincial Center for Disease Control and Prevention. The patients/participants provided their written informed consent to participate in this study.

## Author Contributions

ZM and FZ wrote the first draft of the manuscript. YW revised the first draft of the manuscript. TL and NC made the design. All authors approved the final manuscript.

## Conflict of Interest

The authors declare that the research was conducted in the absence of any commercial or financial relationships that could be construed as a potential conflict of interest. The handling editor LZ declared a shared affiliation, with one of the author NC, at the time of the review.

## Publisher’s Note

All claims expressed in this article are solely those of the authors and do not necessarily represent those of their affiliated organizations, or those of the publisher, the editors and the reviewers. Any product that may be evaluated in this article, or claim that may be made by its manufacturer, is not guaranteed or endorsed by the publisher.
