# Supplementary material for: Network Analysis of Time Use and Depressive Symptoms Among Emerging Adults: Findings From the Guizhou Population Health Cohort Study
Source: Front Psychiatry. 2022 Apr 1;13:809745. doi: 10.3389/fpsyt.2022.809745 (PMC9010560; doi:10.3389/fpsyt.2022.809745)
Supplement: Supplementary file 1 [file Data_Sheet_1.pdf]

Supplementary Material

1 Supplementary Tables

1.1 **Supplementary Table 1** Items used for the assessment of time use and depressive symptoms.

Reference Name	Item
Depressive Symptoms (PHQ-9)	
Anhedonia	Little interest or pleasure in doing things
Sad Mood	Feeling down, depressed, or hopeless
Sleep	Trouble falling or staying asleep, or sleeping too much
Energy	Feeling tired or having little energy
Appetite	Poor appetite or overeating
Guilty	Feeling bad about yourself - or that you are a failure or have let yourself or your family down
Concentration	Trouble concentrating on things, such as reading the newspaper or watching television
Motor	Moving or speaking so slowly that other people could have noticed Or the opposite - being so fidgety or restless that you have been moving around a lot more than usual
Suicide	Thoughts that you would be better off dead, or of hurting yourself in some way
Time use (measured by an adaption of IPAQ-L)	
Heavy Work Activities	Weekly minutes spent on vigorous-intensity physical activities during work, farming, and housework
Moderate Work Activities	Weekly minutes spent on moderate-intensity physical activities during work, farming, and housework
Traffic Time	Weekly minutes spent on walking or cycling for transport
Heavy Leisure Activities	Weekly minutes spent on vigorous-intensity leisure activities such as long-distance running, swimming, and playing football
Moderate Leisure Activities	Weekly minutes spent on moderate-intensity leisure activities such as quick walking and performing Tai Chi
TV Time	Weekly minutes spent on TV watching
Computer Use	Weekly minutes spent on computer use
Video Game	Weekly minutes spent on video game
Sleep Duration	Weekly minutes spent on sleep

PHQ-9: 9-item Patient Health Questionnaire;

IPAQ-L : International Physical Activity Questionnaire.

1.2 **Supplementary Table 2** Estimated edge weights of the network without control variables.

	Heavy Work Activities	Moderate Work Activities	Traffic Time	Heavy Leisure Activities	Moderate Leisure Activities	TV Time	Computer Use	Video Games	Sleep Duration	Anhedonia	Sad Mood	Sleep	Energy	Appetite	Guilty	Concentration	Motor	Suicide
Heavy Work Activities	0	0	0.075	0	0	0	-0.11	0	0	0	0	0	0	0	0	0	0	0
Moderate Work Activities	0	0	0.139	0	0	0	-0.09	0	0	0	0	0	0	0	0	0	0	0
Traffic Time	0.075	0.139	0	0.106	0	0	0	0	0	0	0	0	0	0	0	0	0	0
Heavy Leisure Activities	0	0	0.106	0	0.407	0	0.094	0.064	0	0	0	0	0	0	0	0	0	0
Moderate Leisure Activities	0	0	0	0.407	0	0	0	0	0	0	0	0	0	0	0	0	0	0
TV Time	0	0	0	0	0	0	-0.02	0.016	0	0	0	0	0	0	0	0	0	0
Computer Use	-0.11	-0.09	0	0.094	0	-0.02	0	0.265	0	0.026	0	0	0	0	0.023	0	0.025	-0.01
Video Games	0	0	0	0.064	0	0.016	0.265	0	0	0	0	0	0	0	0	0	0	0
Sleep Duration	0	0	0	0	0	0	0	0	0	0	0	0	0	0	0	0	0	0
Anhedonia	0	0	0	0	0	0	0.026	0	0	0	0.243	0	0.035	0	0.085	0.23	0.08	0.031
Sad Mood	0	0	0	0	0	0	0	0	0	0.243	0	0.267	0.11	0.049	0.178	0.052	0.148	0
Sleep	0	0	0	0	0	0	0	0	0	0	0.267	0	0.278	0.104	0.056	0	0	0
Energy	0	0	0	0	0	0	0	0	0	0.035	0.11	0.278	0	0.293	0	0.151	0	0
Appetite	0	0	0	0	0	0	0	0	0	0	0.049	0.104	0.293	0	0.011	0	0.019	0
Guilty	0	0	0	0	0	0	0.023	0	0	0.085	0.178	0.056	0	0.011	0	0.292	0.107	0.049
Concentration	0	0	0	0	0	0	0	0	0	0.23	0.052	0	0.151	0	0.292	0	0.178	0.082
Motor	0	0	0	0	0	0	0.025	0	0	0.08	0.148	0	0	0.019	0.107	0.178	0	0.262
Suicide	0	0	0	0	0	0	-0.01	0	0	0.031	0	0	0	0	0.049	0.082	0.262	0

Note: All time use behaviors were standardized; P values were presented in parentheses.

1.3 **Supplementary Table 3** Estimated edge weights of the network with control variables.

	Heavy Work Activities	Moderate Work Activities	Traffic Time	Heavy Leisure Activities	Moderate Leisure Activities	TV Time	Computer Use	Video Games	Sleep Duration	Anhedonia	Sad Mood	Sleep	Energy	Appetite	Guilty	Concentration	Motor	Suicide	Female	Age	BMI	Drinking	Smoking
Heavy Work Activities	0	0	0.07	0	0	0	-0.11	0	0	0	0	0	0	0	0	0	0	0	-0.11	0	0	0	0.044
Moderate Work Activities	0	0	0.143	0	0	0	-0.09	0	0	0	0	0	0	0	0	0	0	0	0.108	0	0	0	0
Traffic Time	0.07	0.143	0	0.108	0	0	0	0	0	0	0	0	0	0	0	0	0	0	0	0	0	0	0
Heavy Leisure Activities	0	0	0.108	0	0.407	0	0.089	0.06	0	0	0	0	0	0	0	0	0	0	-0.09	-0.02	0	0	0
Moderate Leisure Activities	0	0	0	0.407	0	0	0	0	0	0	0	0	0	0	0	0	0	0	0	0	0	0	0
TV Time	0	0	0	0	0	0	-0.02	0.019	0	0	0	0	0	0	0	0	0	0	0	0	0	0	0
Computer Use	-0.11	-0.09	0	0.089	0	-0.02	0	0.261	0	0.019	0	0	0	0	0.022	0	0.023	-0.01	-0.03	0	0	0	0.06
Video Games	0	0	0	0.06	0	0.019	0.261	0	0	0	0	0	0	0	0	0	0	0	-0.09	0	0	0	0
Sleep Duration	0	0	0	0	0	0	0	0	0	0	0	0	0	0	0	0	0	0	0	0	0	0	0
Anhedonia	0	0	0	0	0	0	0.019	0	0	0	0.241	0	0.031	0	0.082	0.226	0.077	0.024	0	0	0	0	0
Sad Mood	0	0	0	0	0	0	0	0	0	0.241	0	0.267	0.103	0.051	0.178	0.052	0.148	0	0	0	0	0	0
Sleep	0	0	0	0	0	0	0	0	0	0	0.267	0	0.278	0.098	0.052	0	0	0	0	0	0	0	0
Energy	0	0	0	0	0	0	0	0	0	0.031	0.103	0.278	0	0.295	0	0.149	0	0	0	0	0	0	0
Appetite	0	0	0	0	0	0	0	0	0	0	0.051	0.098	0.295	0	0.011	0	0.011	0	0	0	0	0	0
Guilty	0	0	0	0	0	0	0.022	0	0	0.082	0.178	0.052	0	0.011	0	0.29	0.107	0.051	0	0	0	0	0
Concentration	0	0	0	0	0	0	0	0	0	0.226	0.052	0	0.149	0	0.29	0	0.177	0.078	0	0	0	0	0
Motor	0	0	0	0	0	0	0.023	0	0	0.077	0.148	0	0	0.011	0.107	0.177	0	0.262	0	0	0	0	0
Suicide	0	0	0	0	0	0	-0.01	0	0	0.024	0	0	0	0	0.051	0.078	0.262	0	0	0	0	0	0
Female	-0.11	0.108	0	-0.09	0	0	-0.03	-0.09	0	0	0	0	0	0	0	0	0	0	0	0	0	-0.7	-1.82
Age	0	0	0	-0.02	0	0	0	0	0	0	0	0	0	0	0	0	0	0	0	0	0	0	0
BMI	0	0	0	0	0	0	0	0	0	0	0	0	0	0	0	0	0	0	0	0	0	0	0
Drinking	0	0	0	0	0	0	0	0	0	0	0	0	0	0	0	0	0	0	-0.7	0	0	0	0.418
Smoking	0.044	0	0	0	0	0	0.06	0	0	0	0	0	0	0	0	0	0	0	-1.82	0	0	0.418	0

Note: All time use behaviors were standardized; P values were presented in parentheses.

2 Supplementary Figures

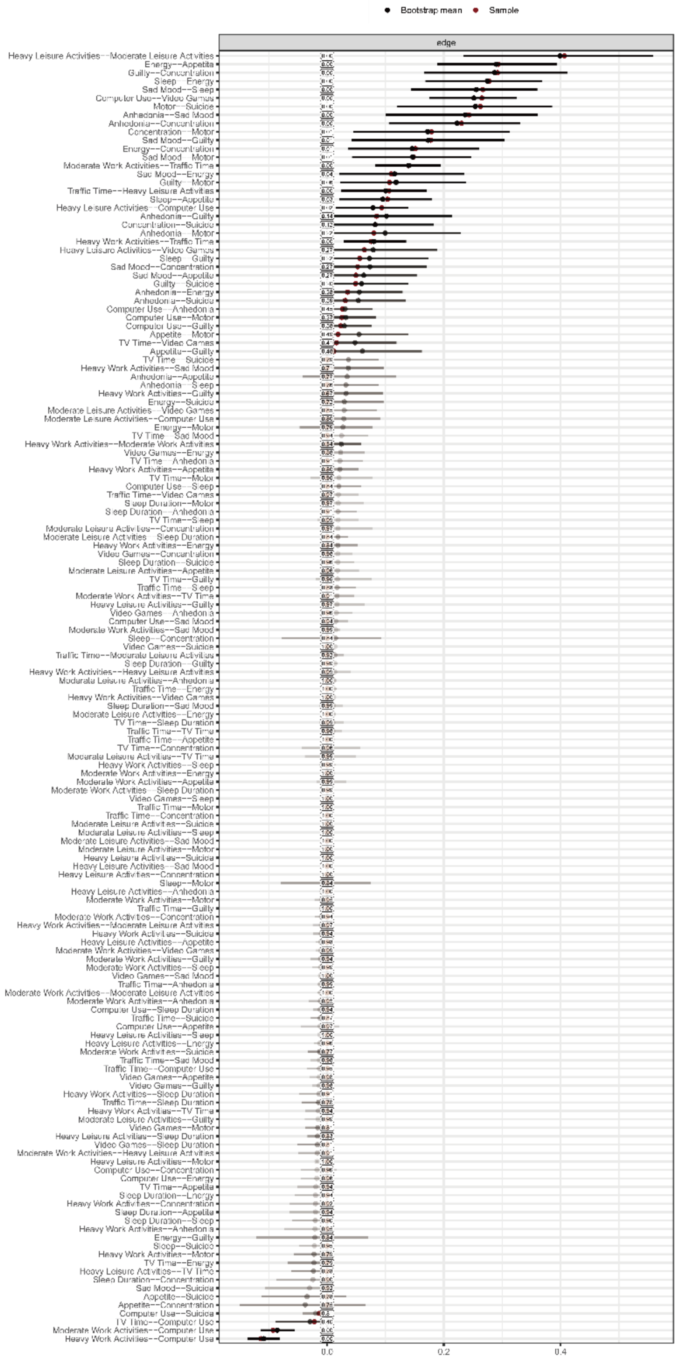

2.1 **Supplementary Figure 1** Accuracy of edge weights of the network without control variables. The x-axle indicates the edge weights, and the y-axle indicates the nodes linked by the edges. The black dots denote the mean value of the bootstrapped edge weights, and the red dots denote the edge weights from current sample. The black lines denote the 95% confidence intervals of the bootstrapped sample.

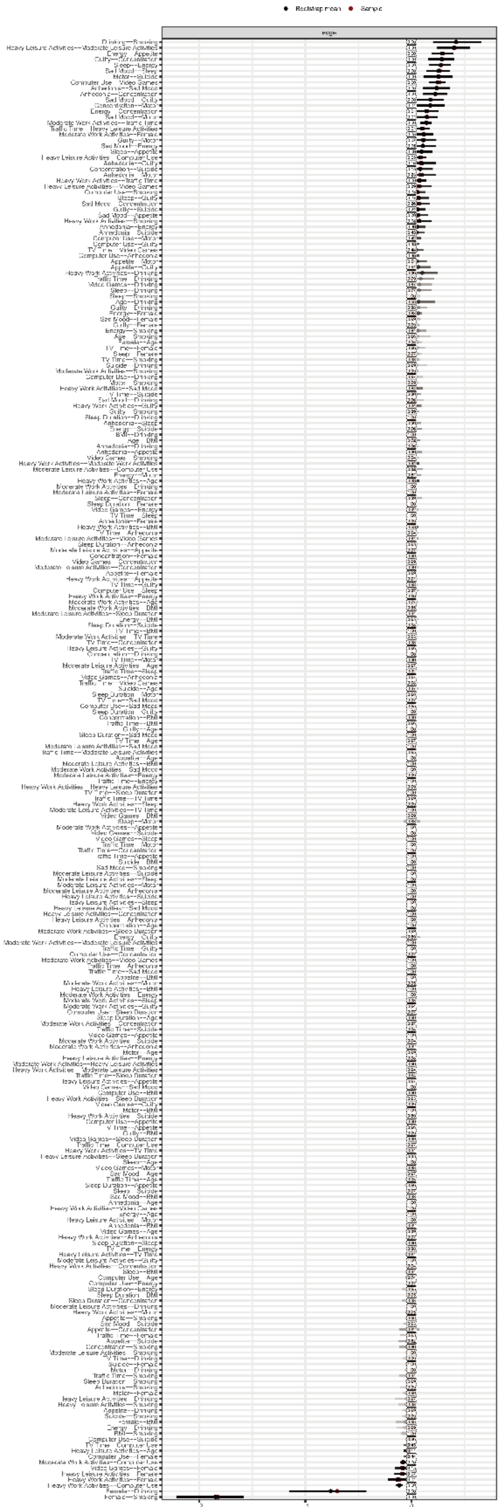

2.2 Supplementary Figure 2 Accuracy of edge weights of the network with control variables. The x-axle indicates the edge **weights,** and the y-axle indicates the nodes linked by the edges. The black dots denote the mean value of the bootstrapped edge **weights,** and the red dots denote the edge weights from current sample. The black lines denote the 95% confidence intervals of the bootstrapped sample.
