# Supplementary figures and images for: Network Analysis of Time Use and Depressive Symptoms Among Emerging Adults: Findings From the Guizhou Population Health Cohort Study
Source: Front Psychiatry. 2022 Apr 1;13:809745. doi: 10.3389/fpsyt.2022.809745 (PMC9010560; doi:10.3389/fpsyt.2022.809745)

● Bootstrap mean ● Sample

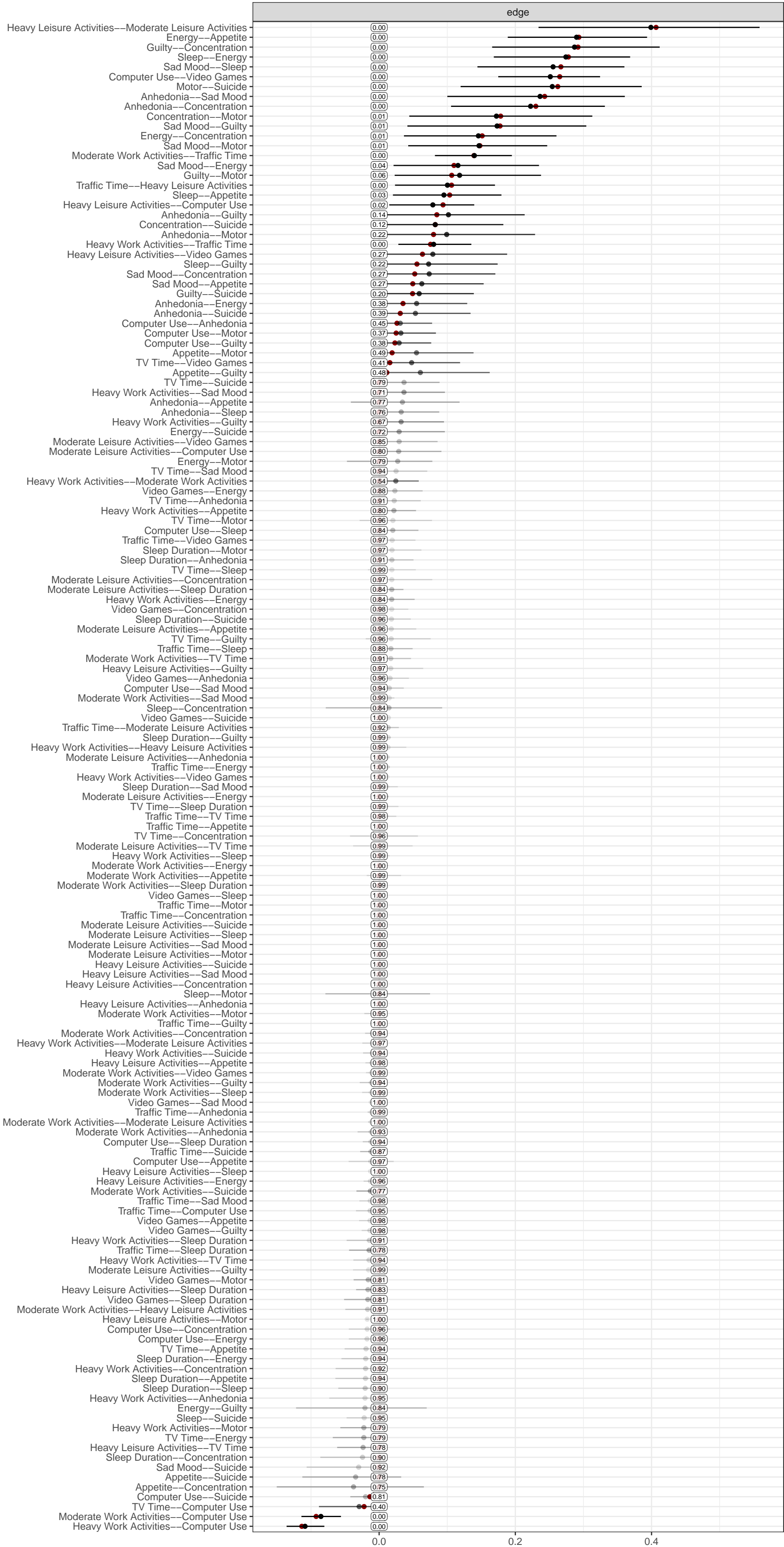

Supplement: Supplementary file 2 [file Image_1.pdf]

● Bootstrap mean ● Sample

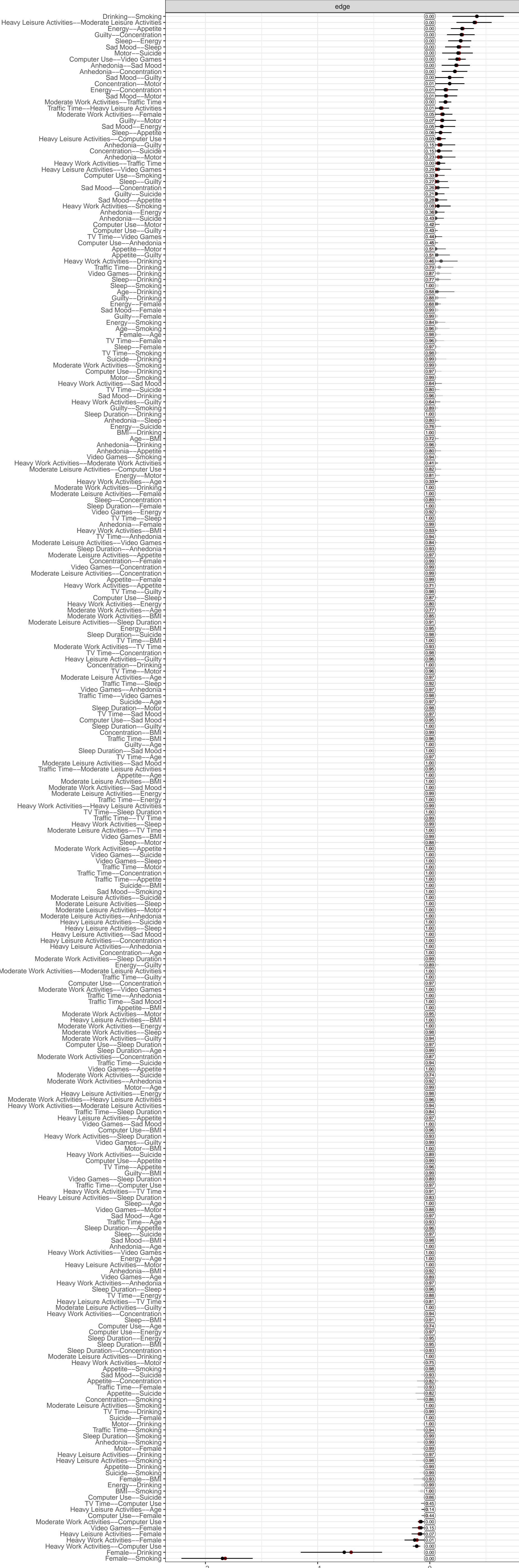

Supplement: Supplementary file 3 [file Image_2.pdf]
